# Serological Survey of Canine Vector-Borne Infections in North-Center Spain

**DOI:** 10.3389/fvets.2021.784331

**Published:** 2021-12-06

**Authors:** Patricia Pérez Pérez, Iván Rodríguez-Escolar, Elena Carretón, José Ángel Sánchez Agudo, Jacob Lorenzo-Morales, José Alberto Montoya-Alonso, Rodrigo Morchón

**Affiliations:** ^1^Zoonotic Infections and One Health GIR, Laboratory of Parasitology, Faculty of Pharmacy, University of Salamanca, Salamanca, Spain; ^2^Instituto Universitario de Enfermedades Tropicales y Salud Pública de Canarias (IUETSPC), Departamento de Obstetricia y Ginecología, Pediatría, Medicina Preventiva y Salud Pública, Toxicología, Medicina Legal y Forense y Parasitología, Universidad de La Laguna, La Laguna, Tenerife, Spain; ^3^Internal Medicine, Faculty of Veterinary Medicine, Research Institute of Biomedical and Health Sciences, University of Las Palmas de Gran Canaria, Las Palmas de Gran Canaria, Spain; ^4^Grupo de Investigación en Biodiversidad, Diversidad humana y Biología de la Conservación, Universidad de Salamanca, Salamanca, Spain; ^5^CIBER de Enfermedades Infecciosas, Instituto de Salud Carlos III, Madrid, Spain

**Keywords:** canine vector borne disease, *D. immitis*, *L. infantum*, GIS, Spain, epidemiology, *E. canis*, *A. platys*.

## Abstract

Various factors are currently causing an increase in vector-borne parasitic diseases at a global scale; among them, some stand out, such as climatic disturbances derived from global change, the increase in movements of reservoir animals, or changes in land made by human activity. In the European continent, there have been an increasing number of epidemiological studies focused on the detection of these diseases, especially in dogs. In Spain, there are few epidemiological studies focused on the evaluation of the biotic and abiotic factors that may influence the distribution, such as climatic zones, orography, or presence of water reservoirs. The aim of this study was to analyze the prevalence and distribution of several canine vector-borne diseases caused by *Dirofilaria immitis, Leishmania infantum, Anaplasma platys*, and *Ehrlichia canis* in the autonomous community of Castilla y León, the largest region of the Iberian Peninsula, providing a geospatial approach based on a geographic information system (GIS) analysis. Blood from a total of 1,475 domestic dogs from the nine provinces of Castilla y León were analyzed. Also, a GIS analysis of the sample locations was carried out, taking into account the most important predictor variables. The prevalence in dogs infected by *D. immitis* was 7.19%, and the seroprevalence by *L. infantum* was 4.61 and 1.56% for *A. platys* and *E. canis*. Most of the infected animals were located in areas with stagnant water, irrigated agriculture, or riverbanks, always close to forest and woodland vegetation. These results indicate that dogs living in Castilla y León should take prophylactic measures to avoid infections.

## Introduction

Canine vector-borne diseases (CVBDs) are caused by several infectious agents, which are transmitted by a wide variety of arthropods, mainly fleas, ticks, mosquitoes, and sandflies. Many of them are zoonotic diseases and are among the most important health problems affecting both domestic dogs and humans worldwide (1). Temperature and humidity are two determining factors that act directly in the establishment and distribution of the vectors and the diseases, as well as other factors derived from human activity such as the increase in the mobility of people and infected animals, or modifications in the landscape, mainly caused by increased irrigated crops or intensive urbanization of new areas. These processes favor the development of some vectors and the infection of the hosts (2–4).

Many of the CVBDs are clinically important, even be fatal, and frequently show unspecific symptoms, which make them difficult to diagnose. These include several zoonotic diseases such as heartworm disease, canine leishmaniasis, and canine anaplasmosis (3–5). In addition, canine ehrlichiosis, although not classified as zoonotic, has been sporadically described in several clinical cases in humans (6, 7).

Nematode *Dirofilaria immitis*, transmitted by culicid mosquitoes, is the causative agent of canine heartworm disease, a chronic disease which can lead to the death of the animal, although many of these animals are asymptomatic and become uncontrolled reservoirs (8). It is a zoonotic parasite which can cause pulmonary dirofilariasis, an asymptomatic infection, but that can be mistaken for lung cancer (2, 9–11). Protozooan *Leishmania infantum*, transmitted by *Phlebotomus* spp., causes canine leishmaniosis and induces a severe disease with serious clinical signs which, if not treated, can lead to the death of the animal. In addition, *L. infantum* can cause cutaneous lesions in humans or visceral leishmaniasis, the latter being a serious condition especially in immunocompromised patients (12). *Anaplasma platys* is the causative agent of canine cyclic thrombocytopenia, while *Ehrlichia canis* is the causative agent of canine monocytic ehrlichiosis. These gram-negative bacteria are mainly transmitted by ticks, and the diseases are characterized by non-specific signs, making diagnosis difficult (13–16). In humans, *E. canis* and *A. platys* have been reported in clinically ill human patients causing from mild, self-limiting febrile illness to fatal infections (6, 7, 17).

In Europe, many epidemiological studies have been published showing a wide variability in the prevalence of the mentioned CVBDs. These differences may be due to the influence exerted by the biotic and abiotic variables of each region as well as the different diagnostic tools used (5, 18). In Spain, few studies have been published on the epidemiological status of these diseases mostly reporting data at a global level without evaluating the influence of environmental factors (i.e., orography, vegetation, climate) of each of the areas studied, especially those focused on *Anaplasma spp*. and *E. canis* (3, 5, 19).

These are CVBDs that are spreading throughout Europe and are considered emerging diseases. Therefore, it is important to evaluate the influence of the possible factors that contribute to this expansion; in this way, more accurate prediction models and measures aimed at stopping this expansion can be studied. The present study is focused on Castilla y León, representing a significant extension of territory, with different orographic and climatic zones depending on the province analyzed, to study the prevalence of the mentioned CVBDs as well as the influence of several biotic and abiotic factors. Also, the implementation of global positioning systems for geolocation of data together with the analysis of the environmental variables of a territory through GIS and spatial correlation models has shown to have a great capacity to understand spatial patterns of distribution of biological events in a territory (20). This ecoinformatic methodology allows the elaboration of predictive models focused on the determination of potential risks of parasitic diseases on a global scope [i.e., (21)] or in specific areas (22, 23).

Therefore, the aim of this study was to deepen the prevalence as well as the geographic distribution patterns of selected causative agents of CVBDs in the autonomous community of Castilla y León (Spain) by using GIS, to understand the causal relationship between the environmental variables and the prevalence of the infections.

## Methods

### Study Area

**Figure 1:**
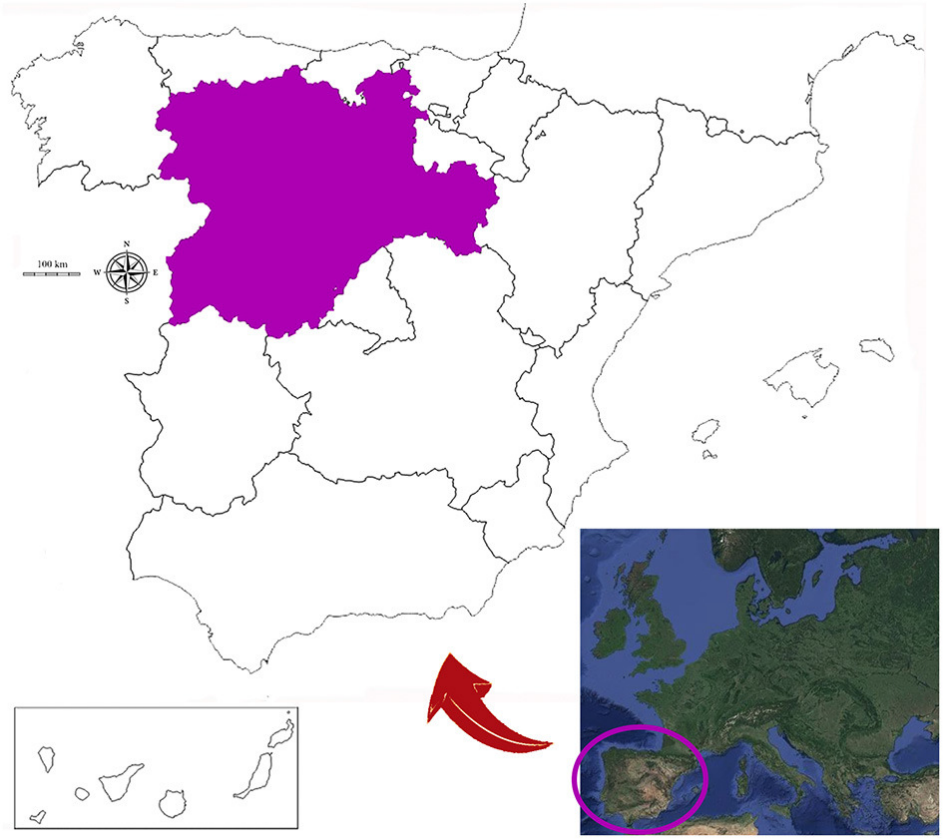
Location of Autonomous Community of Castilla y León, Spain.

**Figure 2:**
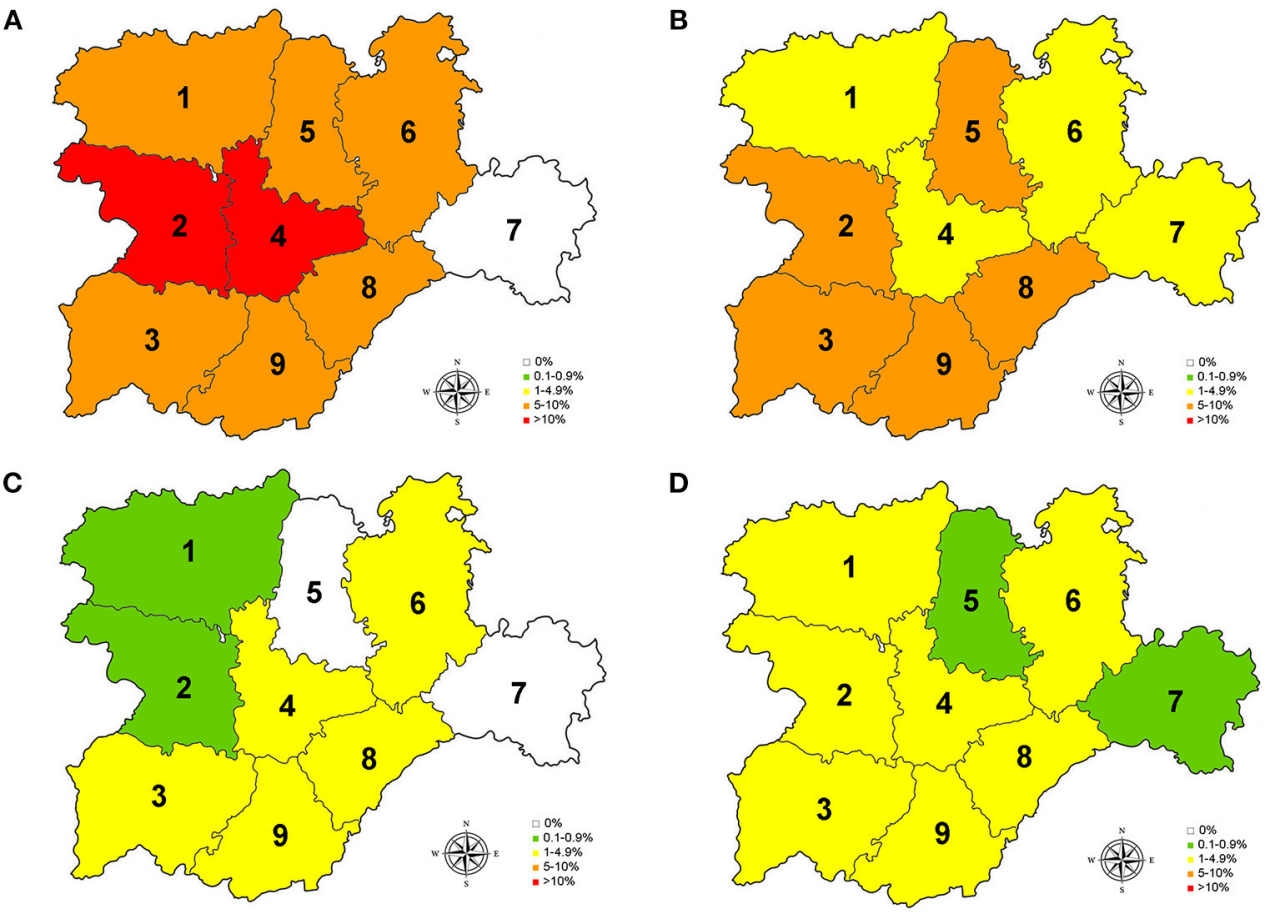
Prevalence for *D. immitis* **(A)**, and seroprevalences for *L. infantum* **(B)**, *A. platys* **(C)**, and *E. canis* **(D)** in the nine provinces of Castilla y León, Spain. 0% (•); 0.1–0.9% (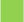); 1–4.9% (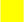); 5–10% (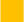); >10 (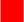).1, León; 2, Zamora; 3, Salamanca; 4, Valladolid; 5, Palencia; 6, Burgos; 7, Soria; 8, Segovia; 9, Ávila.

The Autonomous Community of Castilla y León is located in the northwestern quadrant of the Iberian Peninsula (Figure 1). With an area of 94,224 km^2^, it is the largest region in Spain and one of the largest of Europe, being greater than seven state members of the European Union (Austria, Belgium, Denmark, Holland, Ireland, Luxembourg, and Portugal). The orography of Castilla y León is mainly formed by a plateau with an average altitude of around 800 m above sea level, surrounded by a belt of mountainous reliefs to the north, east, and south, and bordering the west with Portugal. Administratively, it is divided into nine provinces (León, Zamora, Salamanca, Valladolid, Palencia, Burgos, Soria, Segovia, and Ávila) (Figure 2), with León being the largest (15,851 km^2^) and Segovia the smallest (6,921 km^2^) (24, 25).

According to Köppen's climate classification, Castilla y León falls within the continental's Mediterranean climate, presenting long and cold winters, with average temperatures between 3 and 6°C in January, as well as short, hot summers (average temperatures from 19 to 22°C). The average annual rainfall is about 450–500 mm, accentuated in mountain ranges, but with hardly any rainfall during the summer months. Furthermore, due to the great extension and the orographic diversity of this territory, different sub-climates can be distinguished. A large part falls within the temperate with dry or temperate summer (Csb) or temperate with a dry season and temperate summer (Cfb) sub-climates, the average of the warmest month being below 22°C but above 10°C for ≥5 months. In several areas of the central plateau, the sub-climate is classified as temperate with dry or hot summer (Csa) as it exceeds 22°C during the summer, or cold steppe (BSk), with average annual temperatures below 18°C. At high altitudes in the mountain areas, the climate present is cold temperate with average temperatures below 3°C in the coldest months and dry summers (Dsb or Dsc) (26).

According to the map of phytoclimatic series by Rivas Martínez (27), the vegetation of Castilla y León is mainly included in the Mediterranean region, supra-Mediterranean floor, Carpetano-Iberico-Leonesa biogeographic province. The potential forest formations of quercines are predominant in it: holm oaks (*Quercus ilex* subsp. *ballota*), pyrenean oaks (*Q. pyrenaica*), cork oaks (*Q. suber*), and quejigo oaks (*Q. faginea*), which are planted, managed, and regularly pruned, configuring a manmade ecosystem characterized by a savannah-like physiognomy, locally known as “dehesa systems.” These forests cover most of the plains and middle slopes, but some beech (*Fagus sylvatica*) and chestnut forests (*Castanea sativa*) are also present in the mountainous foothills. Sabinas and juniper trees (*Juniperus* sp.) practically complete most of this forest landscape, together with the riverside communities associated with the main rivers and streams that cross the territory.

### Sample Collection

The study included a total of 1,475 blood samples from domestic dogs, collected between September 2019 and December 2020 (Table 1). Samples were collected from dogs undergoing medical examination in 44 veterinary clinics and hospitals, with at least four veterinary clinics for each province of Castilla y León. The participation of clinics and hospitals was voluntary, and samples were collected throughout the duration of the study. Samples came from both owned and shelter dogs. The criteria for inclusion were (a) no previous history of infection, (b) not receiving regular chemoprophylaxis for the studied vector-borne diseases, and (c) owner consent to participate in the survey. Epidemiological data, such as sex, age at presentation to the clinics, and habitat (indoor, outdoor, or indoor/outdoor: at least 1–50% of the time spent outdoors), were recorded.

**Table 1:** Prevalence and seroprevalences of the studied vector-borne infections by sex, age, and habitat in Castilla y León, Spain.

		***D. immitis***		***L. infantum***		***A. platys***		***E. canis***	
	***n***	**+**	**%**	**+**	**%**	**+**	**%**	**+**	**%**
**SEX**									
Male	748	57	7.62	36	4.81	11	1.47	9	1.20
Female	727	49	6.74	32	4.40	12	1.65	14	1.93
**AGE**									
<1 year	68	8	11.76	4	5.88	1	1.47	1	1.47
1–5 years	563	34	6.04	28	4.97	8	1.42	7	1.24
>5–10 years	545	44	8.07	23	4.22	10	1.83	13	2.39
>10-15 years	281	19	6.76	11	3.91	4	1.42	2	0.71
>15 years	18	1	5.56	2	11.11	0	0.00	0	0.00
**HABITAT**									
Indoor	546	24	4.40	22	4.03	6	1.10	2	0.37
Outdoor	646	55	8.51	39	6.04	9	1.39	14	2.17
Indoor/Outdoor	283	27	9.54	7	2.47	8	2.83	7	2.47
**TOTAL**	1,475	106	7.19	68	4.61	23	1.56	23	1.56

*n, number of dogs sampled; +, positive animals; %, percentage of positive animals*.

Blood samples were collected from the cephalic or jugular vein, placed in 3-ml serum tubes, and centrifuged. Serum samples were kept at −20°C until tests were performed. All samples were tested for the detection of *D. immitis* antigens and for the detection of antibodies against *L. infantum, E. canis*, and *A. platys* by using Uranotest Quattro (Uranovet, Barcelona Spain) following the manufacturer's instructions.

### Statistical Analysis

Data were analyzed by using SPSS Base 20.0 software (SPSS Inc./IBM, Chicago, IL, USA). A descriptive analysis of the variables considered was carried out considering the proportions of the qualitative variables. Chi-square and Fisher exact tests to compare proportions were performed. Sex, age, and habitat were considered as variables in the analysis for the autonomous community of Castilla y León and for each province. For the statistical analysis, dogs were grouped into five age groups (<1 year, from 1 to 5 years, from 5 to 10 years, from 10 to 15 years, and >15 years). In all cases, the significance level was established at *p* < 0.05.

### GIS Mapping

A map of the sampling area was constructed using ArcMap v.10.8 (ESRI, 2020 Redlands, CA, USA), including the following layers of relevant environmental information that have been considered to be relevant for the dynamics of the analyzed organisms and their transmission vectors: climate, potential vegetation, and surface waters and surface and edaphic humidity (rivers, lakes, lagoons, irrigated croplands, and parks) (28, 29). Thematic symbols were added for easier interpretation of the map. The canine samples were georeferenced by the location of health centers where the veterinary consultations occurred. Therefore, the points shown on the maps correspond to the centroids of the polygons of the postal codes where the analyses of the dogs were carried out. Inferences were drawn from a proximity analysis between the presence points and the environmental characteristics of the layers used. The coordinate system used was gcs_ETRS_1989.

## Results

Of the studied dogs, 7.72% were positive for one or several causative agents of CVBDs. The overall prevalence of *D. immitis* was 7.19%, and the seroprevalences of *L. infantum, A. platys*, and *E. canis* were 4.61, 1.56, and 1.56%, respectively. Results by sex, age groups, and habitat are shown in Table 1. Results for each of the nine provinces of Castilla y León are shown in Figure 2 and Tables 2–5.

**Table 2:** Numbers of dogs sampled and prevalence of *D. immitis* by sex, age, and habitat in the nine provinces of Castilla y León, Spain.

	**1-León**			**2-Zamora**			**3-Salamanca**			**4-Valladolid**			**5-Palencia**			**6-Burgos**			**7-Soria**			**8-Segovia**			**9-Ávila**		
	**+**	***n***	**%**	**+**	***n***	**%**	**+**	***n***	**%**	**+**	***n***	**%**	**+**	***n***	**%**	**+**	***n***	**%**	**+**	***n***	**%**	**+**	***n***	**%**	**+**	***n***	**%**
**SEX**																											
Male	9	121	7.44	10	65	15.38	6	68	8.82	10	88	11.36	4	50	8.00	7	128	5.47	0	61	0.00	5	110	4.55	6	57	10.53
Female	7	101	6.93	3	44	6.82	4	69	5.80	7	78	8.97	4	62	6.45	17	156	10.90	0	57	0.00	6	114	5.26	1	46	2.17
**AGE**																											
<1 year	2	8	25.00	2	8	25.00	1	9	11.11	0	5	0.00	0	2	0.00	3	22	13.64	0	5	0.00	0	5	0.00	0	4	0.00
1–5 years	5	64	7.81	8	47	17.02	2	41	4.88	4	63	6.35	6	40	15.00	4	97	4.12	0	65	0.00	3	88	3.41	2	58	3.45
>5–10 years	4	82	4.88	3	37	8.11	4	65	6.15	10	55	18.18	1	43	2.33	13	105	12.38	0	37	0.00	4	85	4.71	5	36	13.89
>10–15 years	5	62	8.06	0	16	0.00	3	21	14.29	3	43	6.98	1	25	4.00	4	53	7.55	0	11	0.00	3	45	6.67	0	5	0.00
>15 years	0	6	0.00	0	1	0.00	0	1	0.00	0	0	0.00	0	2	0.00	0	7	0.00	0	0	0.00	1	1	100.00	0	0	0.00
**HABITAT**																											
Indoor	8	65	12.31	4	50	8.00	1	17	5.88	2	63	3.17	1	21	4.76	4	102	3.92	0	83	0.00	2	117	1.71	2	28	7.14
Outdoor	8	154	5.19	6	46	13.04	7	100	7.00	12	60	20.00	3	65	4.62	14	117	11.97	0	21	0.00	1	20	5.00	4	63	6.35
Indoor/Outdoor	0	3	0.00	3	13	23.08	2	20	10.00	3	43	6.98	4	26	15.38	6	65	9.23	0	14	0.00	8	87	9.20	1	12	8.33
**TOTAL**	16	222	7.21	13	109	11.93	10	137	7.30	17	166	10.24	8	112	7.14	24	284	8.45	0	118	0.00	11	224	4.91	7	103	6.80

*n, number of dogs sampled; +, positive animals; %, percentage of positive animals*.

**Table 3:** Numbers of dogs sampled and seroprevalence of *L. infantum* by sex, age, and habitat in the nine provinces of Castilla y León, Spain.

	**1-León**			**2-Zamora**			**3-Salamanca**			**4-Valladolid**			**5-Palencia**			**6-Burgos**			**7-Soria**			**8-Segovia**			**9-Ávila**		
	**+**	***n***	**%**	**+**	***n***	**%**	**+**	***n***	**%**	**+**	***n***	**%**	**+**	***n***	**%**	**+**	***n***	**%**	**+**	***n***	**%**	**+**	***n***	**%**	**+**	***n***	**%**
**SEX**																											
Male	5	121	4.13	4	65	6.15	6	68	8.82	5	88	5.68	3	50	6.00	5	128	3.91	1	61	1.64	3	110	2.73	4	57	7.02
Female	4	101	3.96	4	44	9.09	1	69	1.45	2	78	2.56	7	62	11.29	0	156	0.00	2	57	3.51	8	114	7.02	4	46	8.70
**AGE**																											
<1 year	2	8	25.00	2	8	25.00	0	9	0.00	0	5	0.00	0	2	0.00	0	22	0.00	0	5	0.00	0	5	0.00	0	4	0.00
1–5 years	3	64	4.69	2	47	4.26	2	41	4.88	4	63	6.35	3	40	7.50	2	97	2.06	2	65	3.08	3	88	3.41	7	58	12.07
>5–10 years	1	82	1.22	4	37	10.81	3	65	4.62	3	55	5.45	3	43	6.98	2	105	1.90	1	37	2.70	5	85	5.88	1	36	2.78
>10–15 years	3	62	4.84	0	16	0.00	2	21	9.52	0	43	0.00	3	25	12.00	0	53	0.00	0	11	0.00	3	45	6.67	0	5	0.00
>15 years	0	6	0.00	0	1	0.00	0	1	0.00	0	0	0.00	1	2	50.00	1	7	14.29	0	0	0.00	0	1	0.00	0	0	0.00
**HABITAT**																											
Indoor	4	65	6.15	2	50	4.00	0	17	0.00	3	63	4.76	3	21	14.29	1	102	0.98	0	83	0.00	8	117	6.84	1	28	3.57
Outdoor	5	154	3.25	6	46	13.04	7	100	7.00	3	60	5.00	5	65	7.69	3	117	2.56	2	21	9.52	1	20	5.00	7	63	11.11
Indoor/Outdoor	0	3	0.00	0	13	0.00	0	20	0.00	1	43	2.33	2	26	7.69	1	65	1.54	1	14	7.14	2	87	2.30	0	12	0.00
**TOTAL**	0	222	4.05	8	109	7.34	7	137	5.11	7	166	4.22	10	112	8.93	5	284	1.76	3	118	2.54	11	224	4.91	8	103	7.77

*n, number of dogs sampled; +, positive animals; %, percentage of positive animals*.

**Table 4:** Numbers of dogs sampled and seroprevalence of *A. platys* by sex, age, and habitat in the nine provinces of Castilla y León, Spain.

	**1-León**			**2-Zamora**			**3-Salamanca**			**4-Valladolid**			**5-Palencia**			**6-Burgos**			**7-Soria**			**8-Segovia**			**9-Ávila**		
	**+**	***n***	**%**	**+**	***n***	**%**	**+**	***n***	**%**	**+**	***n***	**%**	**+**	***n***	**%**	**+**	***n***	**%**	**+**	***n***	**%**	**+**	***n***	**%**	**+**	***n***	**%**
**SEX**																											
Male	2	121	1.65	1	65	1.54	1	68	1.47	0	88	0.00	0	50	0.00	2	128	1.56	0	61	0.00	2	110	1.82	3	57	5.26
Female	0	101	0.00	0	44	0.00	1	69	1.45	4	78	5.13	0	62	0.00	2	156	1.28	0	57	0.00	4	114	3.51	1	46	2.17
**AGE**																											
<1 year	0	8	0.00	0	8	0.00	0	9	0.00	0	5	0.00	0	2	0.00	0	22	0.00	0	5	0.00	0	5	0.00	1	4	25.00
1–5 years	1	64	1.56	1	47	2.13	0	41	0.00	1	63	1.59	0	40	0.00	1	97	1.03	0	65	0.00	2	88	2.27	2	58	3.45
>5–10 years	0	82	0.00	0	37	0.00	2	65	3.08	2	55	3.64	0	43	0.00	3	105	2.86	0	37	0.00	2	85	2.35	1	36	2.78
>10–15 years	1	62	1.61	0	16	0.00	0	21	0.00	1	43	2.33	0	25	0.00	0	53	0.00	0	11	0.00	2	45	4.44	0	5	0.00
**>15 years**	0	6	0.00	0	1	0.00	0	1	0.00	0	0	0.00	0	2	0.00	0	7	0.00	0	0	0.00	0	1	0.00	0	0	0.00
**HABITAT**																											
Indoor	0	65	0.00	0	50	0.00	0	17	0.00	2	63	3.17	0	21	0.00	0	102	0.00	0	83	0.00	3	117	2.56	1	28	3.57
Outdoor	1	154	0.65	1	46	2.17	0	100	0.00	1	60	1.67	0	65	0.00	3	117	2.56	0	21	0.00	0	20	0.00	3	63	4.76
Indoor/Outdoor	1	3	33.33	0	13	0.00	2	20	10.00	1	43	2.33	0	26	0.00	1	65	1.54	0	14	0.00	3	87	3.45	0	12	0.00
**TOTAL**	2	222	0.90	1	109	0.92	2	137	1.46	4	166	2.41	0	112	0.00	4	284	1.41	0	118	0.00	6	224	2.68	4	103	3.88

*n, number of dogs sampled; +, positive animals; %, percentage of positive animals*.

**Table 5:** Numbers of dogs sampled and seroprevalence of *E. canis* by sex, age, and habitat in the nine provinces of Castilla y León, Spain.

	**1-León**			**2-Zamora**			**3-Salamanca**			**4-Valladolid**			**5-Palencia**			**6-Burgos**			**7-Soria**			**8-Segovia**			**9-Ávila**		
	**+**	***n***	**%**	**+**	***n***	**%**	**+**	***n***	**%**	**+**	***n***	**%**	**+**	***n***	**%**	**+**	***n***	**%**	**+**	***n***	**%**	**+**	***n***	**%**	**+**	***n***	**%**
**SEX**																											
Male	2	121	1.65	2	65	3.08	1	68	1.47	0	88	0.00	0	50	0.00	0	128	0.00	1	61	1.64	2	110	1.82	1	57	1.75
Female	1	101	0.99	1	44	2.27	1	69	1.45	3	78	3.85	1	62	1.61	4	156	2.56	0	57	0.00	1	114	0.88	2	46	4.35
**AGE**																											
<1 year	1	8	12.50	0	8	0.00	0	9	0.00	0	5	0.00	0	2	0.00	0	22	0.00	0	5	0.00	0	5	0.00	0	4	0.00
1–5 years	0	64	0.00	1	47	2.13	0	41	0.00	1	63	1.59	0	40	0.00	2	97	2.06	1	65	1.54	1	88	1.14	1	58	1.72
>5–10 years	1	82	1.22	2	37	5.41	2	65	3.08	1	56	1.79	1	43	2.33	2	105	1.90	0	37	0.00	2	85	2.35	2	36	5.56
>10–15 years	1	62	1.61	0	16	0.00	0	21	0.00	1	42	2.38	0	25	0.00	0	53	0.00	0	11	0.00	0	45	0.00	0	5	0.00
>15 years	0	6	0.00	0	1	0.00	0	1	0.00	0	0	0.00	0	2	0.00	0	7	0.00	0	0	#¡DIV/0!	0	1	0.00	0	0	0.00
**HABITAT**																											
Indoor	0	65	0.00	1	50	2.00	0	17	0.00	0	85	0.00	0	21	0.00	0	102	0.00	0	83	0.00	1	117	0.85	0	28	0.00
Outdoor	2	154	1.30	2	46	4.35	1	100	1.00	2	61	3.28	1	65	1.54	3	117	2.56	0	21	0.00	0	20	0.00	3	63	4.76
Indoor/Outdoor	1	3	33.33	0	13	0.00	1	20	5.00	1	20	5.00	0	26	0.00	1	65	1.54	1	14	7.14	2	87	2.30	0	12	0.00
**TOTAL**	3	222	1.35	3	109	2.75	2	137	1.46	3	166	1.81	1	112	0.89	4	284	1.41	1	118	0.85	3	224	1.34	3	103	2.91

*n, number of dogs sampled; +, positive animals; %, percentage of positive animals*.

No significant differences were found by sex or age in any of the studied causative agents of CVBDs (Table 1). However, when habitat was assessed, significant differences were observed in the prevalence of *D. immitis* and seroprevalence of *E. canis* between indoor and outdoor dogs (*p* < 0.05) and between indoor and indoor/outdoor dogs (*p* < 0.05). Significant differences between outdoor and indoor/outdoor were observed in results for *L. infantum* (*p* < 0.05). By provinces, only significant differences were observed in the prevalence of *D. immitis* between indoor and outdoor dogs in Valladolid and between indoor and indoor/outdoor dogs in Segovia (*p* < 0.05) (Table 2).

Coinfections were observed in 0.95% of the infected dogs. Of them, 0.34% were infected by *D. immitis* + *L. infantum*, 0.14% by *D. immitis* + *A. platys*, 0.14% by *D. immitis* + *E. canis*, 0.14% by *L. infantum* + *A. platys*, 0.14% by *L. infantum* + *E. canis*, and 0.07% by *A. platys* + *E. canis*. By provinces, coinfections were found in 2.25% of the dogs from León, with 0.9% for *D. immitis* + *L. infantum*, 0.45% for *L. infantum* + *A. platys*, and 0.9% for *L. infantum* + *E. canis*. In Zamora, coinfections were found in 0.73% of the samples, corresponding to *D. immitis* + *A. platys* coinfections. In Valladolid, 0.6% tested positive for *D. immitis* + *E. canis* and 0.6% for *L. infantum* + *A. platys*. In Salamanca, only one dog was infected by *D. immitis* + *A. platys* (0.73%). In Palencia, there were two dogs infected by *D. immitis* + *L. infantum* (1.79%) and in Burgos 0.35% that tested positive for *D. immitis* + *A. platys, D. immitis* + *E. canis*, and *A. platys* + *E. canis*. In Soria, Segovia, and Avila, there were no coinfections in any of the samples analyzed.

Regarding *D. immitis*, the highest prevalences were located in the center and west of Castilla y León, where Csb and Cfb climates were present, while no infected dogs were found in Soria (east). Regarding *L. infantum*, the provinces with the highest seroprevalences were located in the south. On the other hand, seroprevalences for *A. platys* and *E. canis* were low but present in the whole region, except for the absence of *A. platys* in Palencia and Soria. According to climate, positive animals for *L. infantum, A. platys*, and *E. canis* were found in BSk, Cfb, Csa, and Csf sub-climates.

**Figure 3:**
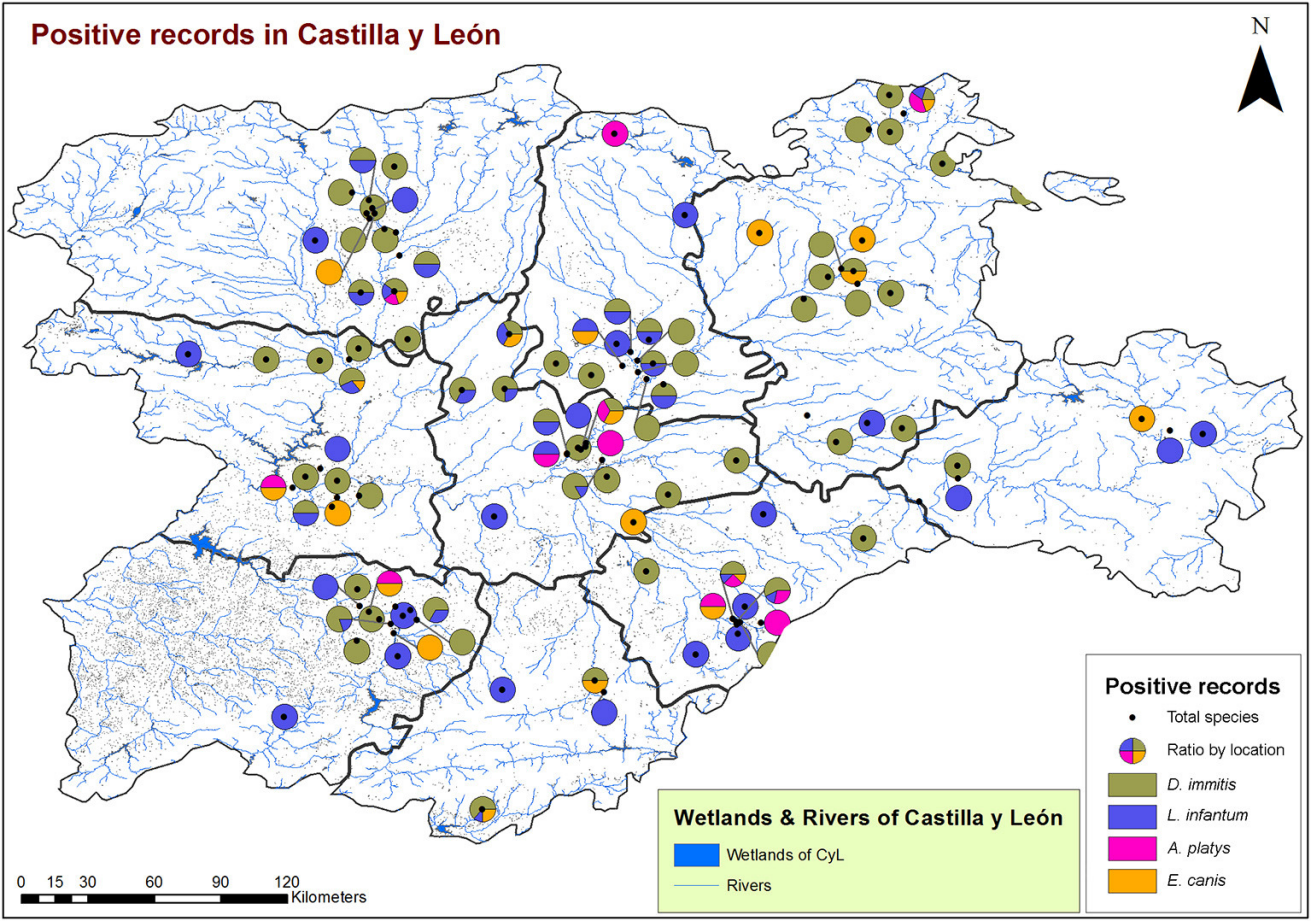
Location of wetlands and rivers and geolocation of dogs infected by *D. immitis* (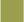), and seroprevalences for *L. infantum* (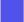), *A. platys* (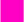), and *E. canis* (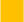) in the nine provinces of Castilla y León, Spain.

**Figure 4:**
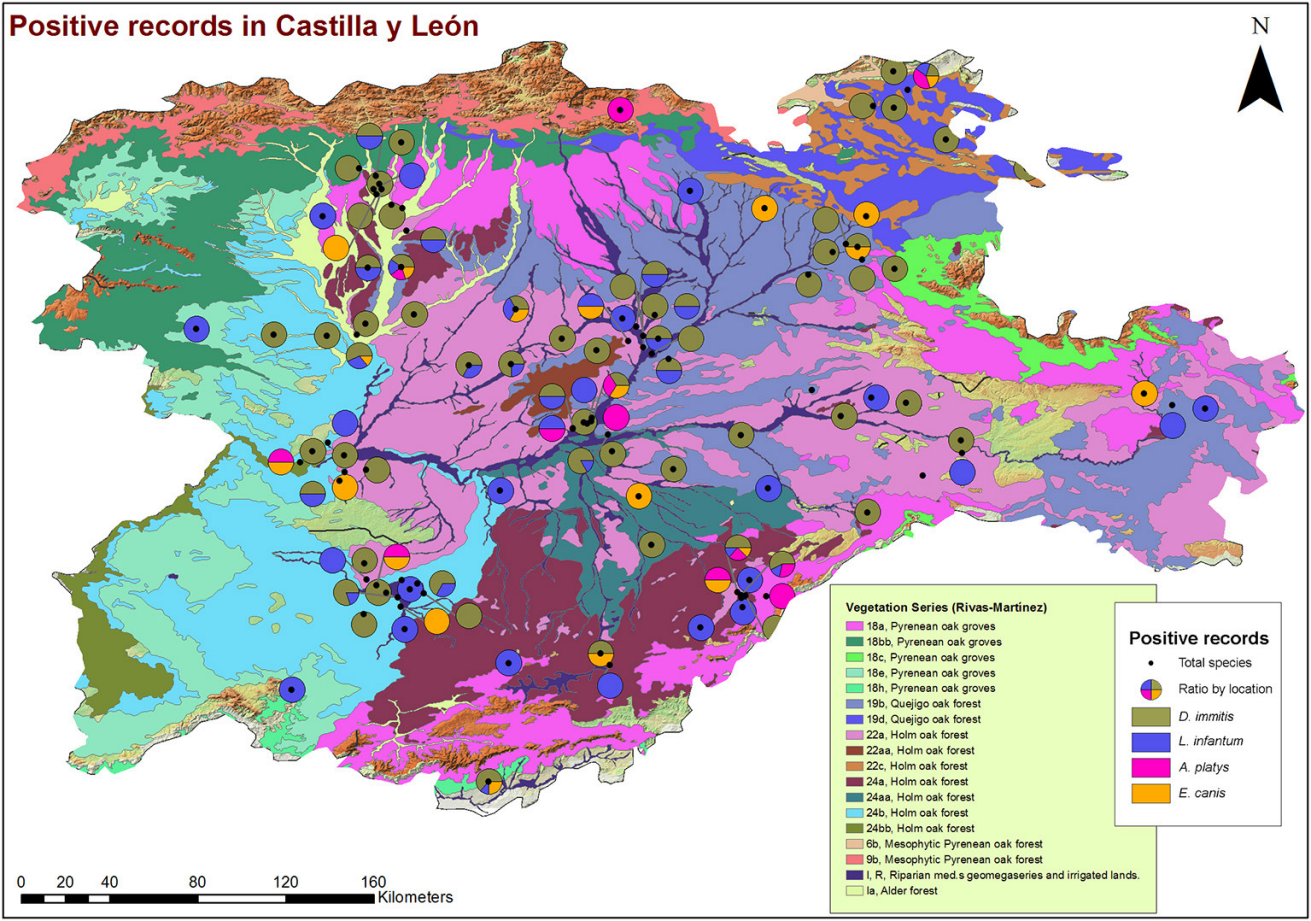
Location of phytoclimatic series and geolocation of dogs infected by *D. immitis* (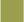), and seroprevalences for *L. infantum* (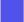), *A. platys* (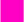), and *E. canis* (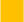) in the nine provinces of Castilla y León, Spain.

From a geospatial point of view (Figures 3, 4), 94.55% of infected animals were located in areas with high edaphic availability of water, as either stagnant water, irrigated agriculture, or river banks, as well as close to forest and groves vegetation, near holm oak, pyrenean oak groves, quejigo oak forest, or riparian forest, all of them mainly found in wet locations with a great abundance of irrigated arable land in the surrounding area. The remaining 5.45% of the infected animals (12/220) did not usually live in these locations but had been in the same or nearby areas with high soil availability of water and similar vegetation. In fact, 6.6% (7/106) were infected by *D*. immitis, 5.88% (4/68) by *L. infantum*, 0% (0/23) by *A. platys*, and 4.34% (1/23) by *E*. canis.

## Discussion

This manuscript shows the prevalence and seroprevalence of four causative agents of CVBDs in Castilla y León, the largest region of the Iberian Peninsula and one of the largest territories of the European Union. The highest positivity detected was that of *D. immitis* (7.19%), followed by *L. infantum* (4.61%), *A. platys*, and *E. canis* (1.56%), although these seroprevalences varied according to the geographical location of the provinces and the distribution of the samples tested.

A slight increase in the global prevalence of *D. immitis* was found when compared to a previous study, which reported a prevalence of 6.23% for Castilla y León (3). Although the data should be considered carefully due to the short time between one study and another, it is necessary to keep a constant record of variations in epidemiology to determine if there is an increasing trend in prevalence, which could confirm the expansion of this parasitosis in Spain (8). In Castilla y León, data at the provincial level only had been previously published for Salamanca, where prevalence decreased from 12.3 to 5.8% within 30 years (30–32); furthermore, a hyperendemic zone was previously reported in Salamanca, characterized by irrigated lands near the riverbank and presence of stagnant water, which showed a decreasing prevalence from 33.3 to 16.7% (8, 30–32). The results of the present study showed a slight increase in Salamanca, which may be due to the spread of the disease as well. The occurrence of canine cardiopulmonary dirofilariasis depends mostly on climatic factors such as temperature and humidity, although changes in land use derived from the human activity (e.g., the increase of irrigated crops), and the management of domestic animals also influences on the presence of the parasite and vectors (33). Although initially relegated to south and east of Spain, studies have confirmed the expansion toward northern and colder areas (3, 8). In addition, the presence of *Cx. pipiens*, widely present in Salamanca, has also been demonstrated to transmit the disease in this province (34). The results of this study confirm the risk of transmission specially associated with the presence of stagnant water, irrigated agriculture, crops, riverbanks, forests, and wet vegetation, as was predicted by a geo-environmental model (35), since infected dogs were located in areas with high or very high risk of infection in all provinces of Castilla y León.

Canine leishmaniosis has traditionally been considered as a disease limited to the Mediterranean basin (36), but several studies have reported an incipient increase in the number of cases in some areas of the north and central regions of Spain (3, 5, 37, 38), although the number of published studies is still low (39). Previous studies carried out in Castilla y León are sporadic and with a low number of samples analyzed. One of them showed an overall seroprevalence of 5.74% (3), and at the provincial level, another study carried out in Valladolid showed a seroprevalence of 5.3% (40). Both showed slightly higher prevalences than those reported in the present study, which could be due to the difference in the sampling process (with lower number of samples) rather than a real decrease in the prevalence, since an increase of this disease is being reported in Spain (3, 5, 39). Although from a geographical and climatic point of view, conditions of Castilla y León are very different and less favorable for the development of sandfly vectors from those in Mediterranean regions considered as endemic for *Leishmania*, the disease is well-established since seropositive dogs were found in irrigated areas, with a range of vegetation prone to vector establishment, and, moreover, cases of human leishmaniosis have been reported in Castilla y León (39), so control measures are necessary. It is possible that this expansion found in heartworm and in leishmaniosis is due to the fact that both diseases are considered not present in Castilla y León and, therefore, are not subject to strict prophylactic measures between veterinarians and owners.

Regarding *A. platys* and *E. canis*, few epidemiological data are available in Spain, but those published report the presence of both infectious agents mainly distributed in the provinces along the Mediterranean, although prevalences between 1 and 4.9% have also been reported in inland and isolated areas of the Iberian Peninsula (3, 4). It seems that the presence in the studied territory has decreased, since previous studies reported global prevalences of 19.2% in 1996 and 2% in 2020 for *E. canis* (3, 40, 41). Similar findings have been seen for *Anaplasma spp*., which previous surveys have reported a global prevalence of 2.74% and prevalence of 19% in Valladolid (3, 40). This decrease is confirmed by the present results. *Ixodes* spp. and *Rhipicephalus sanguineus*, vectors of these diseases, are widely distributed throughout Castilla y León, the Iberian Peninsula, and Europe (42), being mainly located in areas with presence of grass, bushes, or trees. Furthermore, wild animals can act as reservoirs (43, 44), and in those provinces with higher seroprevalences there is a large population of wild animals (wolves, foxes) which in some cases live near houses and villages. In areas bordering the north of the region, where climatic and geographical conditions are much more favorable to vector development, previously reported seroprevalences were notably higher (5.01 and 3.13% to *A. platys* and *E. cani*s, respectively) (45). It seems contradictory since climate changes favor the proliferation of ticks, but it could be due to the use of doxycycline for the treatment of several canine infections, as it is known that the dogs in this study did not receive adequate prophylaxis.

No significant differences were found by sex in this study, similar to other previous studies focused on these diseases such as other studies (3, 11, 19, 46). When age was assessed, no significant differences were observed between age groups, although differences have been observed in other epidemiological studies (4, 5, 11, 47–50). Regarding habitat of the animals, significantly higher prevalences were found in outdoor and indoor/outdoor dogs, as described by other authors (3, 5, 11, 51), since outdoor animals are more exposed to vectors. However, the study showed infected dogs living indoors as well. This is because some arthropods, such as mosquitoes, have access to indoors, and because animals living indoors are not fully enclosed and have a certain amount of access to the outdoors. Therefore, prophylactic measures should be applied to all dogs equally.

The preliminary GIS analysis suggests the existence of patterns of appearance of the infections with certain bioclimatic and environmental variables. The presence of areas with stagnant water, irrigation systems, irrigated agriculture, river banks, and different types of climatologies and vegetation favorable for the development of the vectors detected in the same places where positive dogs have been recorded suggests the existence of patterns of occurrence of the reported diseases with certain bioclimatic and environmental variables (35, 52, 53). The establishment of causal circumstances that can serve to predict risk areas requires a more in-depth study both in the resolution of these variables and in the incorporation of others that can explain in a more precise way the interactions between the parasite, its hosts, and the dispersal vectors. Expanding the methodology through the incorporation of spatial correlation analysis is one of the needs in this epidemiological field.

In conclusion, the results of this epidemiological study show a wide distribution of the evaluated causative agents of CVBDs in Castilla y León, which is very significant given the great geographical extension of the territory. The data obtained reveal the influence of the climate, orography, and presence of water, which will allow to comprehend their evolution in Castilla y León. Given the risk of infection or exposure to pathogens, as their presence in humans in Spain has been described, a close relationship between veterinarians, physicians, and public health administrations under the concept of One *Health* is needed. This would allow effective control measures to be carried out on infected animals and vectors, mainly focused on prophylactic measures to be applied routinely on dogs.

## Data Availability Statement

The raw data supporting the conclusions of this article will be made available by the authors, without undue reservation.

## Ethics Statement

The animal study was reviewed and approved by University of Las Palmas de Gran Canaria. Written informed consent was obtained from the owners for the participation of their animals in this study.

## Author Contributions

EC, RM, and IR-E wrote the manuscript. EC, RM, and JM-A designed the study and obtained funding. PP, IR-E, and JS performed the fieldwork and collected the data. EC, RM, JM-A, and JL-M participated in the revision of the manuscript. All authors participated in the design and production of the figures and have read and agreed to the published version of the manuscript.

## Funding

The study was carried out under the frame of CEVA Salud Animal (Spain), Fundación General de la Universidad de Salamanca, and Agencia de Desarrollo Económico de Castilla y León (cofinanced with FEDER funds-Art. 83).

## Conflict of Interest

The authors declare that the research was conducted in the absence of any commercial or financial relationships that could be construed as a potential conflict of interest.

## Publisher's Note

All claims expressed in this article are solely those of the authors and do not necessarily represent those of their affiliated organizations, or those of the publisher, the editors and the reviewers. Any product that may be evaluated in this article, or claim that may be made by its manufacturer, is not guaranteed or endorsed by the publisher.
